# LncRNA SNHG10 increases the methylation of miR-218 gene to promote glucose uptake and cell proliferation in osteosarcoma

**DOI:** 10.1186/s13018-020-01865-6

**Published:** 2020-08-26

**Authors:** Pan He, Yongqiang Xu, Zhijun Wang

**Affiliations:** Department of Traumatic and Osteopathy, Hunan Provincial People’s Hospital, No. 61 Jiefang West Road, Changsha, 410005 Hunan Province China

**Keywords:** SNHG10, miR-218, Osteosarcoma, Glucose, Methylation

## Abstract

**Background:**

This study aimed to investigate the roles of lncRNA SNHG10 (SNHG10) and miR-218 in osteosarcoma (OS).

**Methods:**

Paired OS and non-tumor tissues were collected from 58 OS patients. The expression of SNHG10 and miR-218 in tissue samples were determined by RT-qPCR. The interaction between SNHG10 and miR-218 was evaluated by overexpression experiment. Methylation-specific PCR was performed to assess the methylation status of miR-218. Glucose uptake in OS cells was analyzed by glucose uptake assay. Cell proliferation was detected by cell proliferation assay.

**Results:**

SNHG10 was upregulated in OS, while miR-218 was downregulated in OS. The expression of SNHG10 and miR-218 were inversely correlated. In OS cells, high glucose induced the upregulation of SNHG10 and downregulation of miR-218. In OS cells, SNHG10 positively, and miR-218 negatively regulated glucose uptake. Overexpression of SNHG10 increased miR-218 gene methylation and decreased the expression of miR-218. In addition, overexpression of SNHG10 also suppressed the inhibitory effects of overexpression of miR-218 on cell proliferation.

**Conclusions:**

SNHG10 increases the methylation of miR-218 gene to promote glucose uptake and cell proliferation in OS.

## Background

As a type of malignancy that causes the production of immature bone, osteosarcoma mainly affects patients younger than 25 years old [1, 2]. In the USA, OS affects 800 to 900 new cases every year [2]. Although a slight higher incidence of OS was observed in China, it only affects 4 out of 1 million people annually [3]. With appropriate surgical operations combined with pre- and post-operative chemotherapy, the overall survival rate of non-metastatic OS patients can reach 77% [4]. However, distant metastasis to other sites of the human body, such as brain and lung, is common in OS [5, 6]. Currently, effective treatments for metastatic OS remain lacking, leading to high mortality rate [7]. Therefore, novel therapeutic approaches are needed.

Targeted therapy, which aims to suppress human diseases mainly by regulating disease-related gene expression, is emerging novel therapeutic approach for cancer treatment [8]. It has been well established that the initiation and progression of OS involve multiple molecular signals and extensive understanding of the functions of these molecules provides novel targets for the treatment of OS [9, 10]. Non-coding RNAs (ncRNAs), such as microRNAs (miRNAs) and long non-coding RNAs (lncRNAs), are not involved in protein synthesis but participate in human diseases, such as OS, by regulating disease-related gene expression [11, 12]. Therefore, lncRNAs and miRNAs are promising targets for OS-targeted therapy. However, their functions in OS are elusive. LncRNA SNHG10 (SNHG10) is a novel oncogenic lncRNA identified in liver cancer [13]. In liver cancer, SNHG10 is overexpressed and forms a positive feedback loop with SCARNA13 to promote carcinogenesis and tumor metastasis [13]. However, the role of SNHG10 in other cancers remains unclear. We performed preliminary RNA-seq analysis and observed its inverse correlation with miR-218. MiR-218 can target GLUT1, a critical player in glucose uptake, to play tumor-suppressive roles [14]. Therefore, SNHG10 may interact with miR-218 to participate in glucose uptake in OS. This study was therefore carried out to investigate the interaction between SNHG10 and miR-218 in OS, with a focus on glucose uptake.

## Methods

### Research subjects

A total of 58 OS patients (35 males and 23 females, 13 years to 40 years old, mean age 26.7 ± 7.0 years old) who were admitted at Hunan Provincial People’s Hospital from May 2016 to May 2019 enrolled. This study was approved by the Ethics Committee of aforementioned hospital before the study. Patients complicated with other clinical disorders or initiated therapies were excluded from this study. All patients were newly diagnosed cases. Patients with recurrent OS were excluded. Based on AJCC staging system, there were 33 cases at stages I and II, and 25 cases at stages III and IV. All patients signed the written informed consent.

### OS tissues and cells

Fine-needle aspiration (FNA) was performed on all 58 OS patients to collect paired OS and non-tumor tissues. All tissues were confirmed by histopathological exam. After that, all tissue samples were immediately subjected to RNA extractions and subsequent experiments.

Human OS cell lines U2OS and MG-63 (ATCC, USA) were used as the cell model of OS. Cell culture medium was composed of FBS (10%) and EMEM medium (90%). Cells were cultivated at 37 °C in a humidity incubator with 5% CO_2_ and 95%. In cases of high glucose treatment, cells were treated with medium containing 10, 20, 30, 40, and 50 mM D-glucose (Sigma-Aldrich) for 48 h before subsequent experiments.

### Transient transfections

Backbone vector expressing SNHG10 was constructed with pcDNA3.1 vector (Invitrogen). Negative control (NC) miRNA and mimic of miR-218 were purchased from Sigma-Aldrich. U2OS and MG-63 cells were harvested at about 85% confluence and were counted after treatment with 0.25% trypsin. After that, vector (1 μg) or miRNA (40 nM) was transfected into 10^8^ cells using lipofectamine 2000 (Invitrogen) following the manufacturer’s instructions. Transfection with empty vector of NC miRNA was also performed to serve as NC group. Control (C) cells were cells without transfections. After transfections, cells were cultivated for further 48 h before subsequent experiments.

### RNA isolation

Total RNAs were extracted from U2OS and MG-63 cells (10^8^ cells) and paired tissue samples (0.02 g) using Ribozol (Sigma-Aldrich). To remove genomic DNAs, all RNA samples were digested with DNase I (Invitrogen) at 37 °C for 2 h. Urea-PAGE gel (5%) was used to evaluate RNA integrity.

### RT-qPCR

RNA samples with a 260/280 ratio close to 2.0 were subjected to reverse transcriptions (RTs) using GoScript™ Reverse Transcriptase kit (A5003, Promega Corporation). With cDNA samples as template, qPCRs were performed using GeneRead qPCR SYBR Green Mastermix (QIAGEN). The expression of SNHG10 was determined with 18S rRNA as internal control. To determine the expression of mature miR-218, all miRNAs were added with poly (A), followed by reverse transcriptions and miRNA qPCRs, which were all performed by All-in-One^TM^ miRNA qRT-PCR Detection Kit (Genecopoeia). All PCRs were repeated three times, and 2^−ΔΔCT^ method was used to process all data.

PCR primers were 5′-GTTGGTCTCTTGGGAGGTAG-3′ (forward) and 5′-CGCCACGACGAACTGCATGC-3′ (reverse) for SNHG10; 5′-CTACCACATCCAAGGAAGC-3′ (forward) and 5′-TTTTCGTCACTACCTCCCC-3′ (reverse) for 18S rRNA. Forward primer of miR-218 was 5′-UGUGCUUGAUCUAACCAUG-3′. Poly (T) was used as reverse primer for both miR-218 and U6. U6 forward primer was from the kit.

### Methylation-specific PCR (MSP)

Cells transfected with SNHG10 expression vector or empty pcDNA3.1 vector were subjected to genomic DNA isolations, which were performed using a Genomic DNA Extraction Kit (ab156900, Abcam). EZ DNA Methylation Lighting Kit (ZYMO research) was used to convert DNA samples, followed by detecting the methylation of miR-218 gene using Taq 2X Master Mix (M0270, NEB). MSP primers were 5′-GTGATAATGTAGCGAGATTTTT-3′ (forward) and 5′-TATAAAAAACTACGTA-3′ (reverse). Routine PCR primers were 5′-GTGATAATGTAGCGAGATTTTC-3′ (forward) and 5′-CACGCAGCTTTCTACA-3′ (reverse).

### Glucose uptake analysis

At 48 h post-transfection, U2OS and MG-63 cells were treated with serum-free EMEM medium for 24 h. After that, cells were further cultivated in medium containing 10 mM glucose for another 48 h. After that, fluorescence-based glucose assay kit (BioVision) was used to measure the levels of intracellular glucose.

### Cell proliferation assay

U2OS and MG-63 cells were transferred to a 96-well plate with 4000 cells in 0.1 ml medium per well. Cells were cultivated at aforementioned conditions, followed by the measurement of OD values at 450 nm every 24 h for a total of 4 days. To monitor cell proliferation, CCK-8 solution (Sigma-Aldrich) was added into each well at 1.5 h before the measurement of OD values.

### Statistical analyses

GraphPad Prism 6 software (GraphPad, USA) was used to analyze data. Three independent replicates were included in each experiment. All data were expressed as mean ± standard deviation (SD) values. Paired *t* test was used to compare paired tissues. Multiple groups were compared by ANOVA Tukey’s test. Repeated measures ANOVA was used to compare different time points. Linear regression was used for correlation analysis. With the median expression levels of SNHG10 or miR-218 in OS tissues as cut-off values, the 58 OS patients were grouped into high and low SNHG10 or miR-218 groups (*n* = 29). Chi-squared test was performed to analyze the associations between the expression levels of SNHG10 or miR-218 and patients’ clinical data. *p* < 0.05 was considered as statistically significant.

## Results

### The expression of SNHG10 and miR-218 was altered in OS

**Fig. 1:**
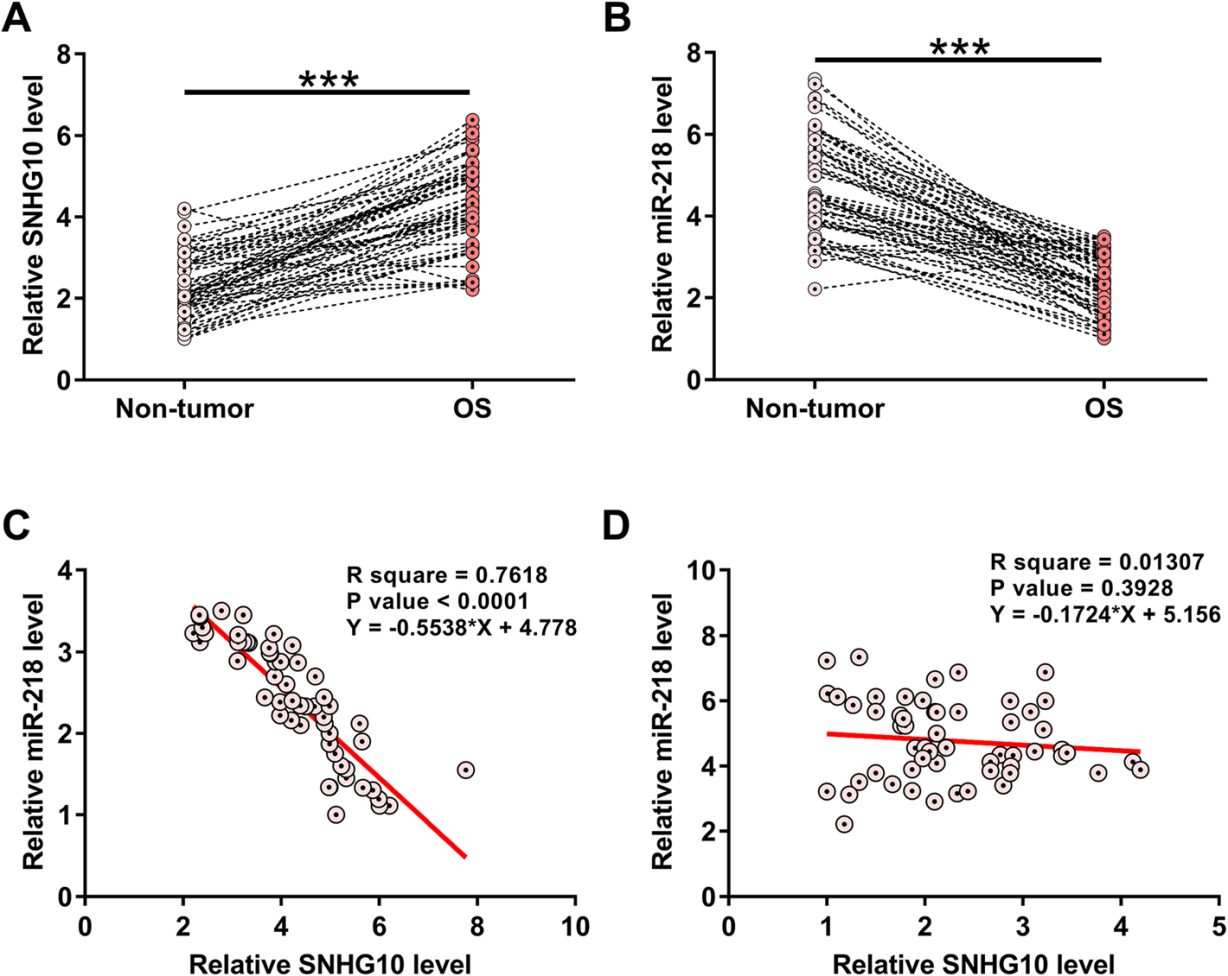
The expression of SNHG10 and miR-218 was altered in OS. The expression of SNHG10 and miR-218 in paired OS and non-tumor tissues from 58 OS patients were determined by RT-qPCR. PCRs were repeated 3 times and mean values were presented. Correlations between SNHG10 and miR-218 across both OS (**c**) and non-tumor (**d**) tissues were analyzed by linear regression. ***p < 0.001

The expression of SNHG10 and miR-218 in paired OS and non-tumor tissues from 58 OS patients were determined by RT-qPCR. Compared to non-tumor tissues, OS tissues exhibited significantly higher expression levels of SNHG10 (Fig. 1a, *p* < 0.001). In contrast, OS tissues exhibited significantly lower expression levels of miR-218 in comparison to that in non-tumor tissues (Fig. 1b, *p* < 0.001). Therefore, altered expression of SNHG10 and miR218 may participate in OS. Correlations between SNHG10 and miR-218 across both OS and non-tumor tissues were analyzed by linear regression. It was observed that the expression of SNHG10 and miR-218 were inversely and significantly correlated with each other across OS tissues (Fig. 1c), but not non-tumor tissues (Fig. 1d). Chi-squared test analysis showed that the expression levels of SNHG10 and miR-218 were closely associated with patients’ clinical stages (both *p* < 0.05), but not patients’ age, gender, BMI, smoking habit and drinking habit (all *p* > 0.05).

### Overexpression of SNHG10 downregulated miR-218 through methylation

**Fig. 2:**
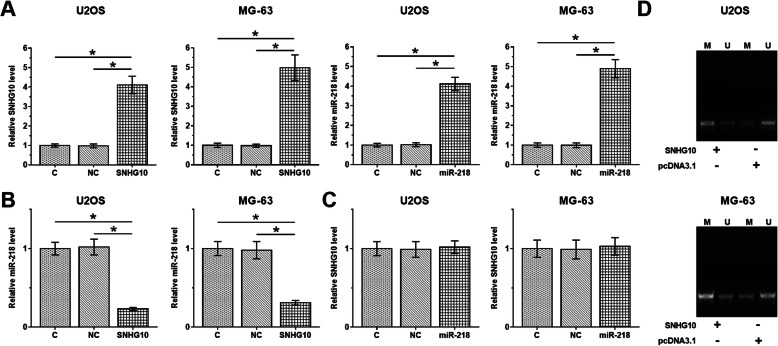
Overexpression of SNHG10 downregulated miR-218 through methylation. Overexpression of SNHG10 and miR-218 in U2OS and MG-63 cells were confirmed by RT-qPCR (**a**). The effects of overexpression of SNHG10 on miR-218 (**b**), and the effects of overexpression of miR-218 on SNHG10 (**c**) were also analyzed by RT-qPCR. MSP was performed to analyze the role of SNHG10 in regulating the methylation of miR-218 (**d**). Three biological replicates were included in each experiment and mean ± SD values were presented and compared. M, methylation; U, unmethylation; *p < 0.05

Overexpression of SNHG10 and miR-218 in U2OS and MG-63 cells were confirmed by RT-qPCR (Fig. 2a, *p* < 0.05). It was observed that overexpression of SNHG10 resulted in downregulation of miR-218 (Fig. 2b, *p* < 0.05), while overexpression of miR-218 did not affect the expression of SNHG10 (Fig. 2c). MSP was performed to evaluate the role of SNHG10 in regulating the methylation of miR-218. Compared to cells transfected with empty vector, cells transfected with SNHG10 expression vector showed significantly increased methylation of miR-218 (Fig. 2d). It was reported that miR-218 was encoded by two genes, miR-218-1 (4p15.31) and miR-218-2 (5q35.1) [15]. MSP was performed on miR-218-2 in this study. Overexpression of SNHG10 showed no significant effects on the methylation of miR-218-1 gene.

### High glucose inducible SNHG10 and miR-218 regulated glucose uptake in OS cells

**Fig. 3:**
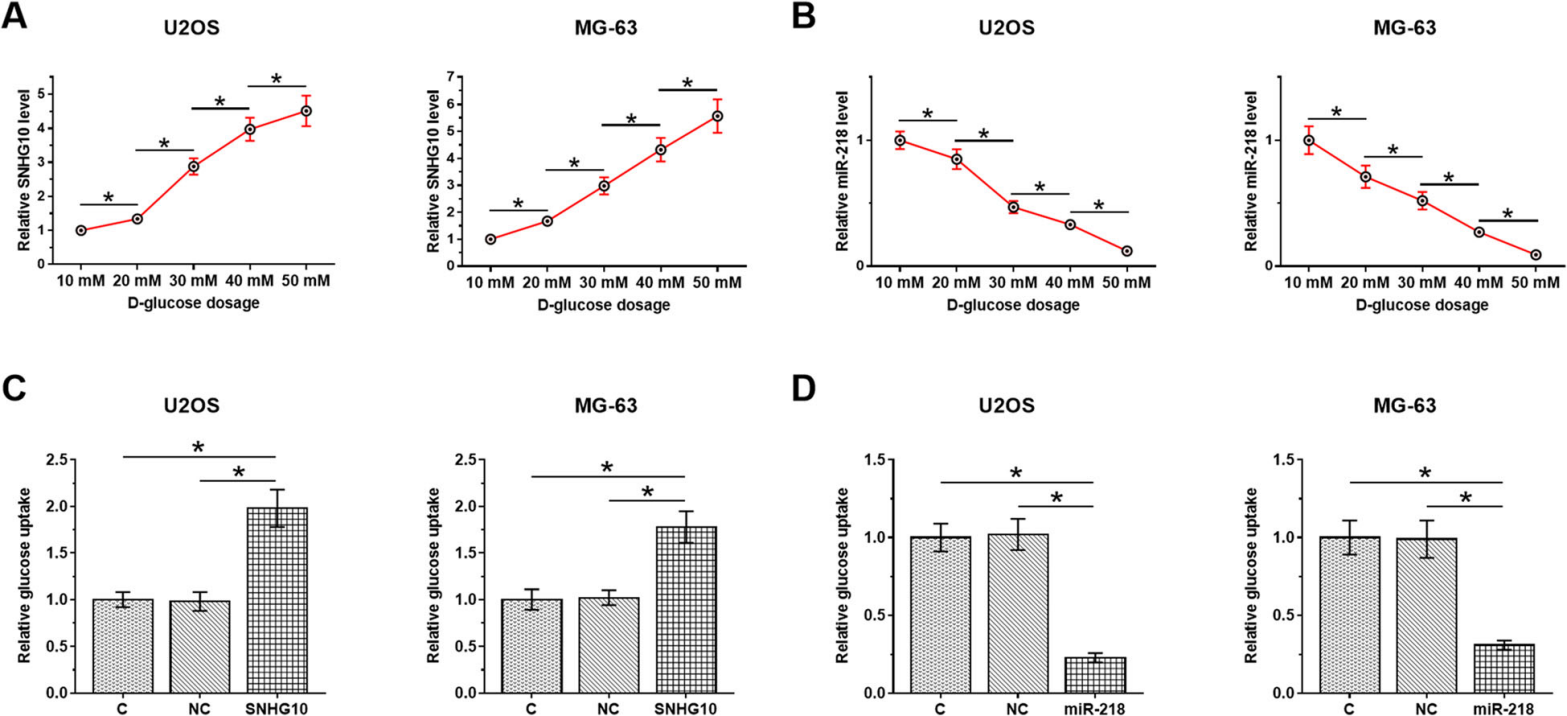
High glucose inducible SNHG10 and miR-218 regulated glucose uptake in OS cells. U2OS and MG-63 cells were treated with medium contaning 10, 20, 30, 40, and 50 mM D-glucose for 48 h, followed by the measurement of the expression levels of SNHG10 (**a**) and miR-218 (**b**) through RT-qPCR. To explore the role of SNHG10 and miR-218 in regulating glucose uptake in OS cells, U2OS and MG-63 cells were transfected with SNHG10 expression vector (**c**) or miR-218 mimic (**d**), followed by glucose uptake. Three biological replicates were included in each experiment and mean ± SD values were presented and compared. *p < 0.05

U2OS and MG-63 cells were treated with medium containing 10, 20, 30, 40, and 50 mM D-glucose for 48 h, followed by the measurement of expression levels of SNHG10 and miR-218 by RT-qPCR. It was observed that high glucose treatment resulted in upregulation of SNHG10 (Fig. 3a, *p* < 0.05) and downregulation of miR-218 (Fig. 3b, *p* < 0.05) in a dose-dependent manner. U2OS and MG-63 cells were transfected with SNHG10 expression vector or miR-218 mimic. Compared to C and NC group, transfection with SNHG10 expression vector increased glucose uptake (Fig. 3c, *p* < 0.05), while miR-218 mimic transfected decreased glucose uptake (Fig. 3d, *p* < 0.05) in OS cells.

### Overexpression of SNHG10 promoted OS cell proliferation through miR-218

**Fig. 4:**
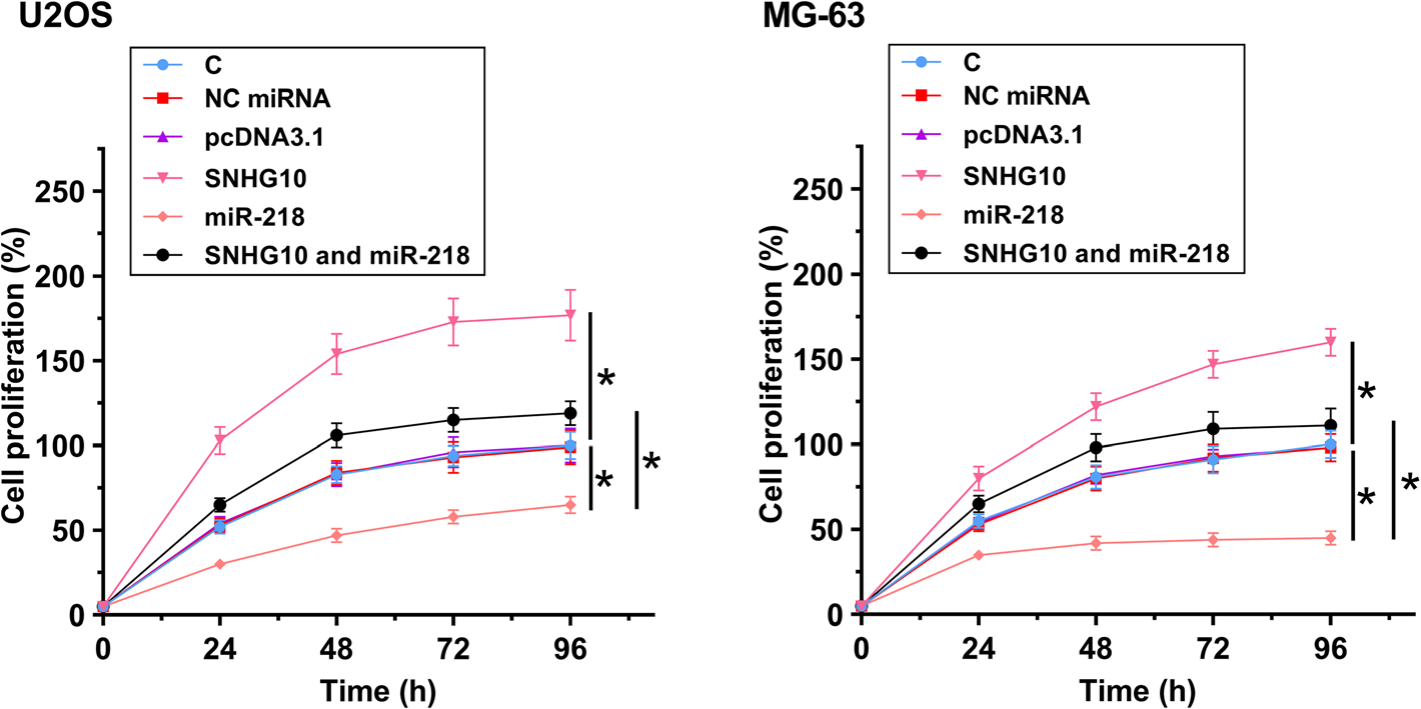
Overexpression of SNHG10 promoted OS cell proliferation through miR-218. The role of SNHG10 and miR-218 in regulating the proliferation of U2OS and MG-63 cells was analyzed by CCK-8 assay. Three biological replicates were included in each experiment and mean ± SD values were presented and compared. *p < 0.05

The role of SNHG10 and miR-218 in regulating the proliferation of U2OS and MG-63 cells was evaluated by CCK-8 assay. Compared to C group, overexpression of SNHG10 promoted cell proliferation, while overexpression of miR-218 inhibited cell proliferation. In addition, overexpression of SNHG10 also suppressed the inhibitory effects of overexpression of miR-218 on cell proliferation (Fig. 4, *p* < 0.05).

## Discussion

In this study, we mainly investigated the interactions between SNHG10 and miR-218 in OS. We found that SNHG10 was upregulated in OS, and it could downregulate miR-218 through methylation to promote the proliferation of OS cells.

The functionality of SNHG10 has only be reported in liver cancer. In liver cancer, SNHG10 was upregulated and predicts poor survival [13]. In addition, SNHG10 regulates the expression of SOX9 to promote cell invasion, proliferation, migration and epithelial–mesenchymal transition in liver cancer [13], suggesting its role as an oncogenic lncRNA. However, the role of SNHG10 in other cancers remains unclear. In this study, we showed that SNHG10 was also upregulated in OS. In addition, overexpression of SNHG10 resulted in increased cell proliferation. Therefore, SNHG10 may also play oncogenic role in OS.

MiR-218 plays tumor suppressive role in different types of cancers including OS [16]. In OS, miR-218 is downregulated and targets BMI1 to suppress OS cell proliferation [16]. Consistently, our study also showed the downregulation of miR-218 in OS and its inhibitory effects on OS cell proliferation. Based on our knowledge, upstream regulators of miR-218 in cancer biology are elusive. In this study we showed that SNHG10 may downregulate miR-218 through methylation in OS. This study is the first to report that SNHG10 can regulate the expression of miRNAs through methylation pathways. However, the expression of SNHG10 and miR-218 were only closely correlated across tumor tissues but not non-tumor tissues. Therefore, the interaction between them may be mediated by certain OS-related factors, which remain to be further identified.

Increased glucose metabolism in cancer cells provides energy for the growth of tumors [17]. Therefore, regulating the glucose uptake may assist the treatment of cancers. In this study, we showed that SNHG10 increased glucose uptake and miR-218 decreased glucose uptake in OS. Therefore, SNHG10 and miR-218 may serve as potential targets for OS treatment by regulating cancer glucose uptake.

## Conclusion

In conclusion, SNHG10 is upregulated in OS and miR-218 is downregulated in OS. In addition, SNHG10 may downregulate miR-218 through methylation to promote the proliferation of OS cells.

## Data Availability

The datasets used and/or analyzed during the current study are available from the corresponding author on reasonable request.

## Abbreviations

SNHG10: lncRNA SNHG10
OS: Osteosarcoma
ncRNAs: Non-coding RNAs
FNA: Fine-needle aspiration

## References

[1] Lindsey BA, Markel JE, Kleinerman ES (2017). Osteosarcoma overview. Rheumatol Ther..

[2] Durfee RA, Mohammed M, Luu HH (2016). Review of osteosarcoma and current management. Rheumatol Ther..

[3] Wu J, Sun H, Li J, Guo Y, Zhang K, Lang C (2018). Increased survival of patients aged 0-29 years with osteosarcoma: A period analysis, 1984-2013. Cancer Med..

[4] Anderson M (2016). Update on survival in osteosarcoma. Orthopedic Clinics of North America..

[5] Onodera H, Yoshida Y, Sakakibara Y, Kono T, Uchida M, Tanaka Y (2011). A case of intracerebral metastasis in osteosarcoma without active pulmonary metastasis. British journal of neurosurgery..

[6] Sueyoshi T, Jono H, Shinriki S, Ota K, Ota T, Tasaki M (2012). Therapeutic approaches targeting midkine suppress tumor growth and lung metastasis in osteosarcoma. Cancer Lett..

[7] Meazza C, Scanagatta P (2016). Metastatic osteosarcoma: a challenging multidisciplinary treatment. Expert Rev Anticancer Ther..

[8] Shaikh AB, Li F, Li M, He B, He X, Chen G (2016). Present Advances and Future Perspectives of Molecular Targeted Therapy for Osteosarcoma. Int J Mol Sci..

[9] Zhou W, Hao M, Du X, Chen K, Wang G, Yang J (2014). Advances in targeted therapy for osteosarcoma. Discov Med..

[10] Lin YH, Jewell BE, Gingold J, Lu L, Zhao R, Wang LL (2017). Osteosarcoma: molecular pathogenesis and iPSC modeling. Trends Mol Med..

[11] Jones KB, Salah Z, Del Mare S, Galasso M, Gaudio E, Nuovo GJ (2012). miRNA signatures associate with pathogenesis and progression of osteosarcoma. Cancer Res..

[12] Li Z, Yu X, Shen J (2016). Long non-coding RNAs: emerging players in osteosarcoma. Tumour Biol..

[13] Lan T, Yuan K, Yan X, Xu L, Liao H, Hao X (2019). LncRNA SNHG10 facilitates hepatocarcinogenesis and metastasis by modulating its homolog SCARNA13 via a positive feedback loop. Cancer Res..

[14] Li P, Yang X, Cheng Y, Zhang X, Yang C, Deng X (2017). MicroRNA-218 Increases the Sensitivity of Bladder Cancer to Cisplatin by Targeting Glut1. Cell Physiol Biochem..

[15] Yang M, Liu R, Li X, Liao J, Pu Y, Pan E (2015). Epigenetic Repression of miR-218 promotes esophageal carcinogenesis by targeting ROBO1. Int J Mol Sci..

[16] Xuan C, Jin M, Gao Y, Xu S, Wang L, Wang Y (2017). MiR-218 suppresses osteosarcoma proliferation by down-regulating BMI1. Int J Clin Exp Med..

[17] Hay N (2016). Reprogramming glucose metabolism in cancer: can it be exploited for cancer therapy?. Nat Rev Cancer..

